# Generating Novel Aroma Phenotypes Using Commercial Wine Samples to Characterize an F1 Population

**DOI:** 10.3389/fpls.2022.894492

**Published:** 2022-06-21

**Authors:** Mani Awale, Connie Liu, Misha T. Kwasniewski

**Affiliations:** ^1^Department of Food Science, The Pennsylvania State University, University Park, PA, United States; ^2^Grape and Wine Institute, University of Missouri, Columbia, MO, United States; ^3^Biosciences Division, Oak Ridge National Laboratory, Oak Ridge, TN, United States

**Keywords:** aroma compounds, interspecific hybrid, F1 population, untargeted metabolomics, GC-MS, phenotyping

## Abstract

Due to their disease tolerance and cold hardy nature, interspecific hybrid grapes are widely grown in the Midwestern and Northeastern United States, with additional interest worldwide in the face of increased abiotic and biotic stresses from climate change. However, the aroma profile of these hybrids is unique and generally less popular in comparison with *Vitis vinifera* grapes. One of the challenges in any phenotyping project is first defining the traits of interest. As wine quality was our ultimate metric of interest, the aroma profile of commercial wines produced from the parents of a breeding population (*Vitis aestivalis* derived ‘Norton’ x *V. vinifera*. ‘Cabernet Sauvignon’) was first assessed for traits of interest. We investigated 11 commercial wines each of Norton, a popular hybrid in Missouri and Cabernet Sauvignon (Cab) for their volatile profiles using the more inclusive metabolomics-based workflow. We then analyzed 21 Norton and 21 Cab grapes from different sites and vintages for the free and bound volatile compounds using HS-SPME-GCMS to validate the differences in wine. The GCMS data was processed using XCMS software to find features that were different between the two cultivars. The two cultivars were found to have differences in their volatile profiles, with 304 features different for wine volatiles, 418 features different for free volatiles, and 302 features different for bound volatiles at 0.05 significance level and with at least a 1.5-fold change between the two cultivars. Those features were used to identify several odor-active compounds in both grapes and wines, including β-damascenone, β-ionone, eugenol, 1,1,6-trimethyl-1,2-dihydronaphthalene (TDN), and methyl salicylate. Some of the identified compounds were higher in Norton than Cab; however, several features were higher in Cab. Using the identified aroma compounds as markers, we phenotyped an F1 population of Norton and Cab. The F1 population was found to be segregating for many aroma compounds with some genotypes demonstrating an even higher concentration of aroma volatiles than either of the parents. Ultimately, using commercially available samples paired with untargeted analysis proved to be an efficient way to determine phenotypes of interest for further analysis and may offer an easy way to choose potential parents with desired traits for breeding.

## Introduction

With the increasing global temperatures, climate change, and an increase in disease and pest populations, the challenge to maintain the current yield and quality of crop plants has led to many breeding programs (Singh and Singh, 2015). While breeding programs may be based primarily on resistance characters, they must also consider the quality-related traits, especially in crops like grapes, where quality above anything drives value. To date, few unique compounds have been found in non-*Vitis vinifera* and hybrids derived from them that can explain the less desirable aroma in interspecific hybrid wines. Many quantitative differences do exist in the identified aroma compounds in those hybrids (Mansfield et al., 2011; Sun et al., 2011; Slegers et al., 2015). Finding meaningful aroma phenotypes or traits that show differentiation in parents and using them in genetic selection of breeding is a critical goal in improving breeding efficiency and the overall quality of selections.

Wine quality is influenced by the volatile compounds such as esters, monoterpenes, and C_13_ norisoprenoids as well as the non-volatile compounds present in the wine including the sugars, organic acids, and phenolic compounds (Ebeler, 2001; Sáenz-Navajas et al., 2012). Volatile aroma compounds and their precursors derived from grapes give a distinct varietal character to the wine produced from different cultivars (Mateo and Jiménez, 2000). The varietal character can sometimes be attributed to a single or few distinct aroma compounds, the interaction of many aroma compounds, and the presence of matrix effects (Ferreira et al., 2000; Villamor and Ross, 2013; Hjelmeland and Ebeler, 2014). Grape-derived aromas are free volatiles that are found in grapes, and bound volatiles are compounds that are glycosidically bound or bound to amino acids and can be released to produce a volatile aroma active compound during vinification (Ebeler, 2001; Ebeler and Thorngate, 2009). Bound volatiles or aroma precursors are not only the reservoirs of desirable wine flavor attributes but may also give rise to some undesirable wine aromas and flavors. These include petrol aroma, foxy aroma, smoke taint, reductive character, and others (Parker et al., 2017). Therefore, it is very important to understand the volatile as well as non-volatile aroma precursors present in grapes that can be potential aroma odorants in the wine (Canuti et al., 2009; Hampel et al., 2014).

In grapevines, *V. vinifera* is the most widely cultivated grapevine species among the 60 species belonging to this genus (This et al., 2006). *V. vinifera* has been used for millennia to produce high-quality wines, but it is susceptible to diseases, pests, and cold. It thus requires frequent pesticide application for commercial production that has questionable economic and environmental sustainability. In the great diversity of *Vitis*, there are species adapted to drought, extreme cold, pest-infested soils, fungal infections, and more; however, they suffer both in fruit quality and by not being self-fertile (Jackson, 2008; Reisch et al., 2012). Interspecific hybrids developed by crossing *V. vinifera*, for its superior fruit quality with other stress-resistant *Vitis* species (Reisch et al., 2012), play an important role primarily in the Eastern and Mid-western United States but also all over the world in face of the atrocities of climate change (Pierre-Louis, 2018; Styles, 2022). These hybrids have lower consumer acceptability than *V. vinifera* wines due to the inheritance of negative flavors from their North American native parents (Biasoto et al., 2014). Some hybrid grape cultivars have been found to contain several undesirable volatile aroma compounds such as *o*-aminoacetophenone and methyl anthranilate (Shure and Acree, 1994), as well as also quantitative differences such as a higher concentration of non-fruity aroma compounds than *V. vinifera* cultivars (Chisholm et al., 1994; Skinkis et al., 2008; Narduzzi et al., 2015; Slegers et al., 2015). Initial research in this area has focused on finding “impact odorants” or singular compounds that could be blamed for hybrids having less preferred wine quality. While some compounds such as methyl anthranilate and *o*-aminoacetophenone were identified in *Vitis labrusca* and its progeny (Shure and Acree, 1994), recent work suggests that the issue with developing wine grapes of high quality is more complex than omitting a handful of aroma compounds. While there have been some targeted studies into the volatile compositions of wines derived from native species (or their progeny), detailed comprehensive analysis of the free and bound volatiles in hybrid grapes and wines and their comparison with the *V. vinifera* grapes and wines have been lacking (Sun et al., 2011; Nisbet et al., 2014; Slegers et al., 2015, 2017). Among the recent work that looked at the volatile compounds in interspecific hybrids, no unique compounds were found, which maybe in part due to the methods in which the mass spectral data was analyzed. In targeted analysis, only a few significant compounds are measured and thus quantified, making it possible to miss compounds or complex relationships between them (Tikunov et al., 2005). With non-targeted analysis, there is an expanded possibility of finding new compounds, as all mass spectral data is mined for differences, but it also can be exceedingly difficult to sort the vastly expanded data set (Commisso et al., 2013; Roullier-Gall et al., 2014; Narduzzi et al., 2015; Schrimpe-Rutledge et al., 2016). To properly express all-important flavor differences between *V. vinifera* wine and a less preferred interspecific hybrid wine, it may be better to cast a bigger net and use a metabolomics-based approach. This will make sure that potentially important information or markers are not omitted in the interest of a simple answer. Ultimately, this can allow identification of complex trends and variability among populations that would have been overlooked using a targeted analysis for a limited number of aroma active compounds.

Norton is a *Vitis aestivalis*-derived grape cultivar that is favorable for humid regions with long growing seasons and has demonstrated good resistance to many fungal diseases like powdery mildew, downy mildew, Botrytis bunch rot, and black rot, as well as Pierce’s disease and phylloxera (Reisch et al., 1993; Sapkota et al., 2015). Although Norton does not possess the characteristic foxy odor of many hybrid grapes, the high malic acid and phenolics in the Norton grapes possess many problems in winemaking that negatively impact the wine quality. It also has a very distinctive aroma that would not be mistaken for *V. vinifera*. To the best of our knowledge, this is the first study that used a metabolomics-based approach to profile the volatiles important in Norton wines and berries. Cabernet Sauvignon is a widely cultivated *V. vinifera* grape cultivar that is widely accepted for high-quality red wine. However, it is not cold-tolerant and is susceptible to many diseases. Several grape breeders have made crosses between Norton and Cabernet Sauvignon with the hope of producing a cultivar with the wine quality of Cabernet Sauvignon and the environmental and biological tolerances of Norton. These efforts lack much information on the flavor differences between the varieties (for example, are the differences due to key impact odorants or just variations in the concentration of common compounds), and the likelihood of a cross between these two will have on improving both resistance and wine quality. Additionally, while flavor is among the key factors of grape and wine quality, little is known about the genetic basis of aroma (Doligez et al., 2006; Lin et al., 2019). Therefore, this study aims to identify aroma differences in Norton and Cabernet Sauvignon using the commercial wine samples and then validate those compound differences using the diverse grape samples. We also aim to assess the application of identifying the compound differences in a breeding population that can be utilized in finding markers for expedited breeding. This is immediately useful, as by knowing what compounds are aromatically important it is possible to leverage previous research to adjust viticultural and winemaking practices such as the impact of cluster exposure and leaf removal on C_13_ norisoprenoids (Kwasniewski et al., 2010; Awale et al., 2021).

## Materials and Methods

### Chemicals

All aroma standards other than 1,1,6-trimethyl-1,2-dihydronaphthalene (TDN) were purchased from Sigma-Aldrich (St. Louis, MO, United States) at >98.8% purity. TDN synthesized from alpha-ionone was donated by Dr. Gavin Sacks Lab at Cornell University (Kwasniewski et al., 2010). A C_7_–C_30_ hydrocarbon mixture, used for the determination of Kovats’ retention indices (RI), was obtained from Sigma-Aldrich. Sodium chloride was purchased from Fisher Chemicals (Fair Lawn, NJ, United States). Rapidase Aroma Revelation (AR2000) was purchased from Creative Enzymes (Shirley, NY, United States). Ultrapure water (Type 1 water) was prepared using the ELGA Lab Water PURELAB Classic (High Wycombe, United Kingdom). L-Tartaric acid (99%) was obtained from Sigma-Aldrich.

### Sample Preparation for Wine

A total of 22 single variety commercial wines procured at a local market were used for initial assessment of cultivar aroma differences. We sampled 11 wines each of Norton and Cabernet Sauvignon from different states (Missouri and California) and countries (the United States and Australia). To prevent oxidation of the wine, the wine bottles were opened in a nitrogen-filled glove box to create an inert atmosphere and 5 ml aliquots of the wine were added to 2 g of sodium chloride (NaCl) in a 20-ml amber SPME glass vial to inactivate the enzymes and improve headspace partitioning (Canuti et al., 2009). An internal standard solution was added at a volume of 10 μl to each vial to make up the final concentrations of 0.1 mg/L 4-methyl-2-pentanol, 0.05 mg/L 3-octanone, and 0.05 mg/L 2-octanol from the standard stock solutions. The vials were then sealed and analyzed using the headspace solid-phase microextraction gas chromatography mass spectrometry (HS-SPME-GC-MS) method outlined below. All 22 samples were run in two replications in a randomized order in two sequential ordered blocks. Internal standards were added for both metabolic and quantitative analyses to ensure all conditions remain comparable throughout the experiments (Mizuno et al., 2017). Additionally, blanks were run after every 5–6 runs to prevent sample carryover. The quality and consistency of the data were monitored by the following methods: (1) for untargeted analysis by the area of the internal standards not varying from the mean by more the 20% (a disadvantage of SPME is its high run to run variability) and (2) for untargeted feature analysis and semi-quantitative analysis of identified compounds all duplicates results maintained a CV below 15% utilizing the internal standards to account for a run to run SPME extraction variation.

### Fruit Samples

We sampled 21 Norton and 21 Cabernet Sauvignon grapes for this experiment. The Norton and Cabernet Sauvignon grapes used in this experiment were harvested during 2012–2016 from Rocheport, MO; Mountain Grove, MO; and Blacksburg, VA. Representative triplicate samples were used and included a range of “mature” fruit final brix ratings to allow for the variability known to exist in the F1 population (14.9 to 23.7 degrees brix) to be analyzed. The samples were flash-frozen in liquid nitrogen after harvesting and transported to the laboratory in dry ice where they were stored at −80°C until used for the analysis in 2018.

### Sample Preparation for Free Volatiles

Sample preparation was performed following the method by Canuti et al. (2009) and Hampel et al. (2014) for free and bound volatiles respectively. For both free and bound volatiles, 60 g of the frozen grape berries were thawed and then ground using a handheld grinder. To prevent oxidation of the berries, 50 μl ascorbic acid (200 g/L stock concentration) was added before grinding. The ground grapes were centrifuged at 11,000 rpm using Eppendorf 5840R for 15 min at 4°C. The supernatant was discarded and the grape solids (mostly skins) were further extracted overnight with sodium phosphate extraction buffer (0.1 M sodium phosphate extraction buffer of 13% ethanol by volume adjusted to a pH of 4.5) in a circular shaker (Labnet Orbit 1000, Edison, NJ, United States) at 105 rpm for 16 h overnight followed by centrifugation for 15 min at 11,000 rpm (model 5804 R, Eppendorf AG, Hamburg, Germany) to separate the solids from the extract. The supernatant was pipetted off and used for further free and bound volatile analysis.

In a 20 ml amber SPME glass vial, 5 ml of the extract was taken and 50 μl of internal standards were added to make a final concentration of 0.01 mg/L 2-octanol and 0.1 mg/L of 4-methyl-2-pentanol, followed by the addition of 2 g NaCl per vial. The samples were then processed for volatile aroma compounds using the HS-SPME-GC-MS. All samples were run in a randomized order in two replicate analysis blocks. Internal standards were added for both metabolic and quantitative analyses to ensure all conditions remain comparable throughout the experiments (Mizuno et al., 2017). Additionally, blanks were run after every 5–6 runs to prevent sample carryover.

### Sample Preparation for Total/Bound Volatiles

Glycosidically bound (or glycosidase releasable) volatiles were extracted using a method adapted from Hampel et al. (2014) and Awale et al. (2021). Briefly, a stock Rapidase enzyme solution (250,000 mg/L) (lot no 91542020P) was prepared using 2.5 g of Rapidase in 10 ml of DI water. In a 20 ml glass amber vial, 5 ml aliquots of the prepared supernatant were spiked with 20 μl of the Rapidase enzyme solution to yield a final enzyme concentration of 1,000 ppm and 50 μl of the internal standard solution to yield a final concentration of 0.01 mg/L 2-octanol and 0.1 mg/L of 4-methyl-2-pentanol. The glass vials were sealed and incubated in a 45°C water bath for 4 h. The vials were then cooled to ambient temperature in a 25°C water bath for 10 min to prevent headspace volatilization. A total of 2 g of NaCl was added to the sample vial to inactivate the Rapidase enzyme. The samples were then processed for volatile aroma compounds using the HS-SPME-GC-MS method. Values are either reported as total or bound whereby the free volatiles were subtracted from the total observed after enzymatic digestion.

### Headspace Solid-Phase Microextraction Gas Chromatography Mass Spectrometry Analysis

Extraction was conducted based on a method of Hampel et al. (2014). Briefly, a 65-μm polydimethylsiloxane (PDMS)/divinylbenzene (DVB) 1-cm SPME fiber was used for extraction (Supelco, Bellefonte, PA, United States). The samples (grape extracts and wines) in 20 ml amber glass vials were preincubated for 15 min at 45°C to ensure consistent temperature during extraction. The fiber was exposed for 45 min at 45°C in the headspace above the sample prior to GC-MS analysis. All samples were agitated at 500 rpm during extraction.

The HS-SPME-GC-MS system consisted of a PAL autosampler (Varian, Palo Alto, CA, United States) mounted on an Agilent 7890B gas chromatograph (Santa Clara, CA, United States) with Agilent 5977A mass selective detector (MSD). The SPME fiber was desorbed in the inlet at 250°C for 14.7 min, with the inlet in a splitless mode for 2 min (inlet glass liner/SPME direct, 0.75 mm I.D., Supelco, Bellefonte, PA, United States), after which spit flow was turned on (50 mL/min) for the remainder of the GC-MS run. No carry-over was observed between samples with blanks run routinely. The samples were run in two replications randomly in two sequential ordered blocks, with the features averaged. GC oven program was used as described in Awale et al. (2021). The MSD was operated in SIM/Scan mode, with SIM mode utilized for the quantification of β-damascenone during targeted analysis (121 m/z; 190 m/z, range 40–250 m/z; 6.4 scans/s).

### Data Processing Using Untargeted Metabolomics Analysis

The raw data acquired by Masslynx from GC-MS were converted to. cdf (common data format) data using the OpenChrome software. Then, the .cdf files were uploaded to XCMS online^1^ as a paired batch job, where peak detection, retention time correction, chromatogram alignment, metabolite feature annotation, statistical analysis, and putative identification were performed using the default parameters (Tautenhahn et al., 2012; Awale et al., 2021). The results were downloaded from the XCMS online on 19 February 2018. The features (intensity of a given *m/z* at a certain time) that are significantly different using *t*-test (FDR adjusted *p*-value < 0.05; Benjamini and Hochberg, 1995) at 0.05 level and have higher or equal to two-times fold change were filtered and used for further analysis. Principal component analysis (PCA) was performed in log-transformed and autoscaled data using MetaboAnalyst 3.0 (Montreal, QC, Canada; Xia and Wishart, 2016).

### Identification of Compound and Confirmation

Compound identification and confirmation were performed based on the method of Awale et al. (2021). Briefly, the significant features identified using ANOVA and PCA loadings were grouped based on their retention time, and the compounds represented by the features were identified using the NIST MS Search v2.2, NIST 14 Mass Spectral Library database (Scientific Instrument Services, Ringoes, NJ, United States). Additionally, linear RI calculated using the Kovats’ equation from a sequence of linear hydrocarbons from C7 to C30 were matched with the NIST data and literature. Confirmation was achieved by comparing mass spectra obtained from the sample with those from the pure standards injected in the same conditions. A selection of identified compounds of interest was then quantified using calibration curves for each compound at five different concentration levels. For compounds whose standards were not available, semi-quantitative analysis was done using the internal standards 2-octanol or 4-methyl-2-pentanol, which were used as internal standards.

### Screening F1 Population Using Identified Volatiles

A cross between Norton and Cabernet Sauvignon was done in 2005 and planted in the Missouri State Fruit Experiment Station vineyard in 2007 (Adhikari et al., 2014; Sapkota et al., 2015). Since grapevines are heterozygous, the F1 population is segregating and thus can be used as a mapping population to study the genetics of the traits. In the fall of 2016, the berries from 90 F1 genotypes (2 replication each) were harvested and then processed for the free and bound volatile analysis of the berries. Free and bound compounds were quantified in the F1 population using the same GC-MS method as used in berry samples with glycosidase-released compounds used as a proxy for fermentation released aromas (Hampel et al., 2014).

### Data Analysis

We used an integrated metabolomics workflow for data analysis as used by Awale et al. (2021). The data were analyzed using MetaboAnalyst 3.0 (Xia and Wishart, 2016) and R version 4.1.2 statistical software. In MetaboAnalyst, the features with more than 50% missing values were removed and the remaining missing values were replaced by a small value (half of the minimum positive value in the original data). Data filtering using an interquartile range was then performed to remove features arising from the baseline noises and to remove features that do not change throughout the treatments that are unlikely to be used for further analysis. Then sample normalization was performed to account for the differences among the sample, and for the wine data, normalization was done using a reference sample which is the 2-octanol internal standard area. Then, the data was log-transformed and auto-scaled (mean-centered and divided by the standard deviation of each variable) and then used for further statistical analysis. The autoscaling method was used to remove any variation comprised during the analysis (such as a loss of instrumental sensitivity) of an original HRMS peaks list. Univariate statistics, such as a *t*-test, was performed for exploratory data analysis to identify the potentially significant features to discriminate the treatments under study. To consider the type II error with repeated tests, FDR correction was applied and FDR-adjusted *p*-value < 0.05 was considered (Benjamini and Hochberg, 1995). The PCA was performed with significant features, which is a type of unsupervised method.

## Results

**FIGURE 1:**
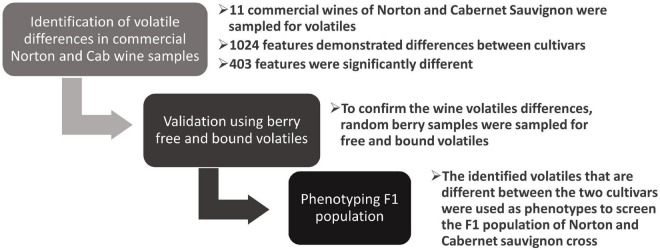
Research workflow: step 1: the supermarket bought commercial wines were phenotyped to identify differences in volatiles, step 2: the random berry samples of the cultivars were phenotyped for free and bound volatiles to confirm the differences in wine, step 3: using the confirmed compounds in both wines and berries, the F1 population of Norton and Cab were phenotyped.

**FIGURE 2:**
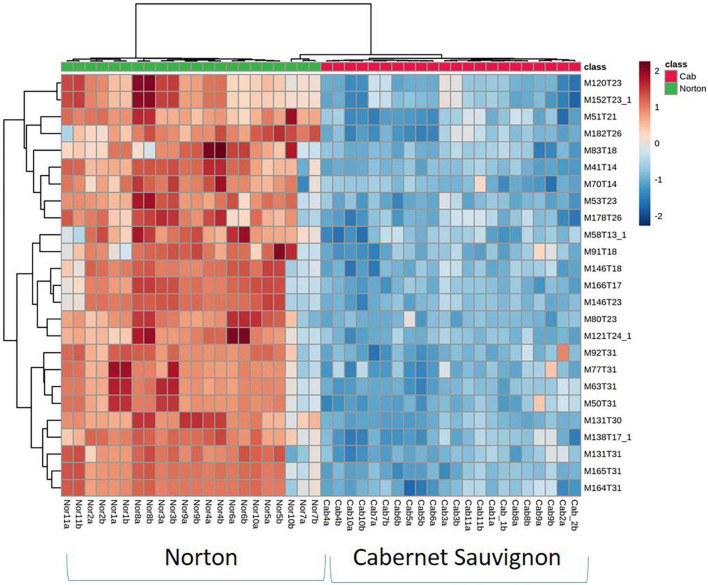
Heatmap of the top 25 most influential features for differentiating wine volatiles between Norton and Cabernet Sauvignon. While only top contributors are shown the heatmaps were generated using all the features. The rows in the heatmap represent features [M (m/z),T (time in minutes)] and the columns indicate sample categories. The colors of the heatmap cells indicate the abundance of compounds across different samples. The color gradient, ranging from dark blue through white to dark red, represents low, middle, and high abundance of a compound, respectively.

Wine samples purchased from the supermarket were used to identify differences between the two grape cultivars, and the differences were then validated using random berry samples from different locations, vintages, and different states of ripening (Figure 1). From these wines, we identified more than a thousand significant features between the two cultivars. The feature is a molecular entity with a definite mass and retention time, designated as M (mass/charge) and T (time in minutes) (Schrimpe-Rutledge et al., 2016). These features were then subjected to multivariate analysis, outlined in detail below, to find the compounds that were driving differences between the two cultivars. The PCA scores plot indicated that there was a distinct separation in the volatile profile between wines of the two cultivars despite no effort being made to control for winemaking interventions (Figure 2). We also observed similar differences in free and total volatiles between the berry samples of the two cultivars. The significant feature differences (FDR adjusted *p*-value < 0.05, fold change > 1.5) were then used to tentatively identify 165 unique compounds using the NIST library, 34 of which were confirmed and quantified using authentic standards and calibration curves. Among the compounds quantified in wines and berries, 14 were identified and quantified in an F1 mapping population.

### Feature Extraction

#### Wine Volatiles

**FIGURE 3:**
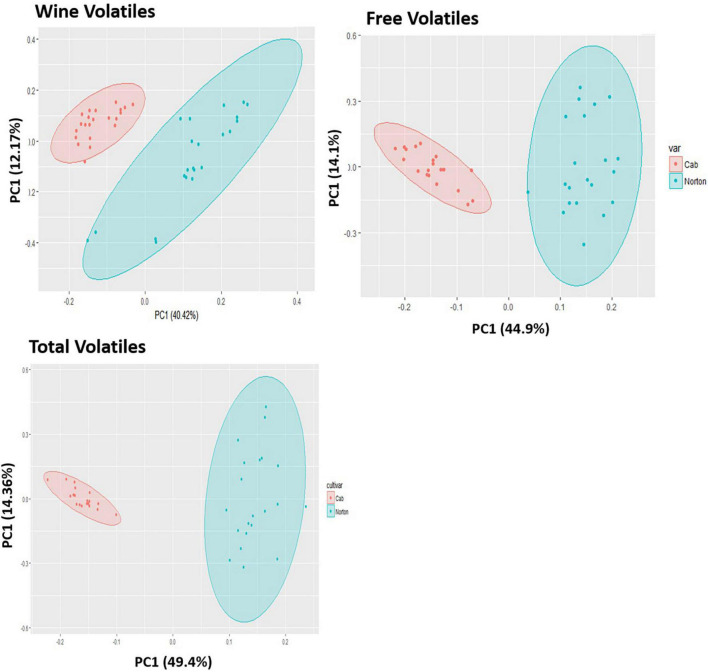
Principal components analysis (PCA) scores plot for the distribution of **(A)** wine volatiles, **(B)** free volatiles, **(C)** total volatiles features in Norton and Cabernet Sauvignon (Cab) grapes and wines. The ellipses show 95% confidence interval. There is a separation based on cultivar for free, total, and wine metabolic features.

Of 1,064 identified features that characterized the populations, 403 were significantly different at a 5% level of significance and had greater than the 1.5-fold change between Norton and Cabernet Sauvignon (Supplementary Table 1). There were 207 features present at higher intensities in Cabernet Sauvignon wines, whereas 196 features were higher in Norton wines. The Norton and Cabernet Sauvignon wines generally had a consistent difference in feature intensity across the wines (Figure 2). To reduce the dimensionality of the data and better represent most of the variation of the data through a few unrelated dimensions, unsupervised PCA was performed using the 403 significantly different features between Norton and Cabernet Sauvignon. PCA analysis using the significant features between the two cultivars indicated that the commercial wines of these two cultivars confirmed the differences in the volatile composition (Figure 3A). PC1 explained 40.42% of the variation, mostly the variation between two cultivars, whereas PC2 explained 12.17% of the variation. The differences were driven by many features which were later identified to be methyl salicylate, β-damascenone, phenylethyl alcohol, and eugenol among others. The two cultivars showed clear isolation in terms of the wine volatiles and did not show any differences in terms of the winery, vintage, as well as the location. Norton wines had a greater distribution compared to Cabernet Sauvignon which might be due to stylistic variation in winemaking styles of Norton.

#### Free Volatiles

To validate the differences observed between the two cultivars in wine, which were fruit-derived, we performed berry volatile analysis on berry samples from different locations and maturity. Similar to the wine findings, Cabernet Sauvignon showed higher intensity of 240 metabolic features in comparison to Norton, whereas Norton grapes had a higher intensity of 178 features (Supplementary Table 2). The PCA scores plot demonstrated differences in the free volatile composition and proportion between two cultivars, in which PC1 explained 44.9% of the variation and PC2 explained 14.1% of the variation. PC1 demonstrated separation due to cultivar in aroma profile whereas PC2 indicated variation between samples within the cultivar (Figure 3B). PCA loadings show that there were several features which were later identified to be methyl salicylate, nerol, and TDN among others that were driving the differences between the two cultivars, and many of these features were also shared with the wine samples. The two cultivars formed two separate groups without any overlapping, which indicates that the free volatiles in Norton and Cabernet Sauvignon are very different. Additionally, we also performed PCA using ripeness (as determined by brix) and location (figures not shown) and found no distinct separation based on either location or state of maturity.

#### Total/Glycosidically Bound Volatiles

In our study, the total volatiles represents the total of all volatiles present in the berries, including free volatiles and volatiles released from precursors due to enzymatic action. The only features that should increase would be due to the enzymatic release of glycosidically bound compounds, though there also could be overlap with free volatiles, for those compounds unchanged by glycosidase. The Norton and Cabernet Sauvignon exhibited 302 significant features differences for total volatiles among the 793 features identified (Supplementary Table 3), among which 215 features were present in higher intensity in Cabernet Sauvignon than Norton. Similarly, 87 features were significantly higher in intensity in Norton grapes. The PCA using the significant features demonstrated no overlap between the two groups indicating that Norton and Cabernet Sauvignon are very different in terms of the total volatile composition (Figure 3C). PC1 explained 49.4% of the variation and demonstrated mostly separation due to genotype, whereas PC2 explained around 14.38% of the variation between samples within the genotype (Figure 3C). These separations were driven by many features that differentiate the two cultivars and also shared with free and wine volatiles. Again, these cultivars did not show distinct differences in terms of location or varying degrees of maturity. The separation between the two cultivars was not as strong as what was observed in wine or berries, but it did yield some significant features differentiating the cultivars not observed in the other samples.

### Identification of Compounds From Significant Features

To understand the biological significance of the feature differences, compound identification was performed. Using the NIST library database, comparing RI with that of literature, and comparing the mass spectra with the standards available in the laboratory, we were able to tentatively identify a total of 165 unique compounds among which 83, 41, and 70 putative compounds are free, bound, and wine volatile, respectively (Supplementary Table 4). Of these, 13 compounds were common in the free, bound, and wine volatiles samples. The compounds identified in this way were further screened to make sure they were likely to be sample-derived and not artifacts of sample preparation or analysis. For the compounds whose authentic standards were available, proper calibration and internal standards were used to minimize the run-to-run variation. Compounds from a wide range of classes such as fatty acid alcohols, norisoprenoids, terpenes, esters, terpinols, and acids were found to vary between the two cultivars (Table 1).

**TABLE 1:** Mean concentration (μg/L) and standard error of mean (SEM) of free volatiles, total volatiles, and wine volatiles in Norton and Cabernet Sauvignon.

Name	Odor threshold^a^	Free volatiles				Total volatiles				Commercial wine volatiles			
		Norton		Cabernet Sauvignon		Norton		Cabernet Sauvignon		Norton		Cabernet Sauvignon	
		Mean	SE	Mean	SE	Mean	SE	Mean	SE	Mean	SE	Mean	SE
β-Caryophyllene	64	0.14	0.06	0.07	0	0.14	0.02	0.08	0.01	nd	nd	nd	nd
p-Cymene		0.15	0.06	0.01	0.01	0.45	0.25	0	0	5.12	0.57	1.9	0.15
Terpinolene	14 mg/L	0.29	0.01	0.26	0.02	0.79	0.02	0.82	0.02	0.26	0.02	0.38	0.02
D-Limonene	10	0.49	0.25	0.15	0.03	3.72	1.58	0.44	0.09	nd	nd	nd	nd
Methyl hexanoate		1.42	0.11	1.53	0.15	1.05	0.27	0.92	0.29	nd	nd	nd	nd
β-Ionone	0.03	1.68	0.2	2.14	0.18	2.04	0.18	2.7	0.29	nd	nd	nd	nd
β-Cyclocitral	0.15	1.73	0.22	1.79	0.13	1.17	0.18	1.17	0.13	nd	nd	nd	nd
β-Linalool	6–25.2	3.47	0.24	1.66	0.08	6.74	0.35	3.35	0.24	9.87	0.93	9.58	0.94
β-Damascenone	0.05	4.06	0.3	2.37	0.14	22.71	1.69	15.78	1.86	7.26	0.8	4.34	0.39
Ethyl hexanoate	14	4.6	0.87	4.87	1.25	2.53	0.54	4.42	1.95	nd	nd	nd	nd
TDN	2	7.35	0.67	2.66	0.24	6.53	0.57	2.75	0.4	2.13	0.27	1.54	0.13
1-Nonanol	50	10.78	0.18	1.68	0.11	9.28	0.31	1.51	0.15	3.2	0.24	3.59	0.19
Nerol	400	11.66	0.66	1.26	0.09	7.13	0.29	6.62	0.17	nd	nd	nd	nd
Methyl salicylate	40	18.15	2.27	0.08	0.02	9.08	0.88	0	0	4.11	0.6	0.93	0.08
Benzaldehyde	5,000	27.43	2.62	15.58	2.14	19.54	3.4	9.03	3.11	31.99	2.87	15.62	1.64
Eugenol	3	51.25	6.15	0.47	0.04	60.34	4.58	0.38	0.04	nd	nd	nd	nd
β-Phenylacetaldehyde	250	136.45	16.43	57.07	5.16	86.98	16.88	50.69	5.17	nd	nd	nd	nd
1-Hexanol	500	227.19	77.51	225.61	48.63	342.55	108.12	235.44	60.43	283.42	32.93	257.38	13.94
2-Phenylethyl alcohol	750–11,000	278.4	24.17	86.2	14.05	456.28		444.55		13.21	2.57	33.89	4.06

*^a^Odor thresholds were reported in μg/L, unless specified (Ferreira et al., 2000; Escudero et al., 2007; Slegers et al., 2015).*

### Quantitation of the Important Volatile Compounds

**FIGURE 4:**
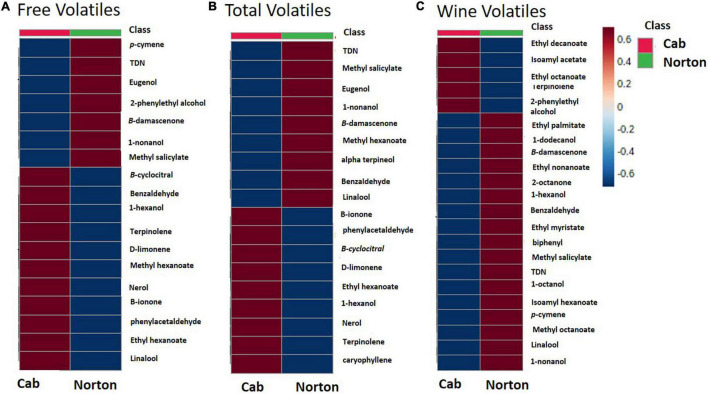
Heatmaps of the abundance of the volatiles in Norton and Cabernet Sauvignon (Cab) **(A)** free volatiles, **(B)** total volatiles, and **(C)** wine volatiles. Heatmaps were created using average quantitative values based on 2-octanol IS. The rows in the heatmap represent volatile compounds and the columns indicate cultivars. The colors of the heatmap cells indicate the abundance of compounds across different samples. The color gradient, ranging from dark blue through white to dark red, represents low, middle, and high abundance of a compound, respectively.

**FIGURE 5:**
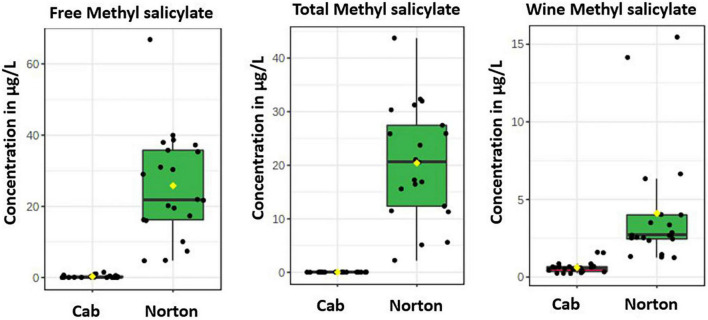
Boxplots showing the distribution of methyl salicylate in Norton and Cabernet Sauvignon (Cab) **(A)** free volatiles, **(B)** total volatiles, and **(C)** wine volatiles. The *y*-axis denotes concentration in μg/L obtained semi-quantitatively relative to 2-octanol.

**FIGURE 6:**
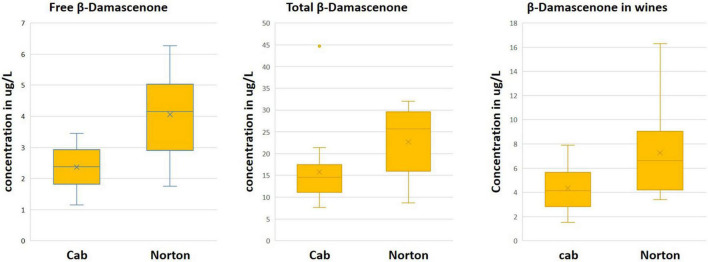
Boxplot showing the distribution of β-damascenone in Norton and Cabernet Sauvignon (Cab) **(A)** free, **(B)**, total, and **(C)** wine volatiles. The *y*-axis denotes concentration in μg/L.

Based on the results from the untargeted compound identification, we quantified 22 volatile compounds for wine and 20 compounds for free and total volatiles. The compounds quantified and their concentration in wine, free and total, are shown in Table 1 and Supplementary Table 5. PCA using the quantified compounds along with the biplot also demonstrated separation based on the cultivars for all free, total, and wine volatiles (Supplementary Figure 1). As with the features, the volatiles that are driving the differences were methyl salicylate, β-damascenone, TDN, 2-phenylethyl alcohol, and eugenol. Some volatile compounds such as β-ionone, methyl hexanoate, terpinolene, ethyl hexanoate, and β-cyclocitral were found in higher concentrations in Cabernet Sauvignon as free volatiles, whereas Norton has a higher amount of eugenol, methyl salicylate, 2-phenylethyl alcohol, and 1-nonanol as free and total volatiles (Figures 4A,B and Table 1). Compounds such as methyl salicylate and β-damascenone are present in higher concentrations in wine and berries as both free and total volatiles (Figures 5, 6). The compounds that were significantly different between the two cultivars were used as stable aroma phenotypes to phenotype the F1 breeding population.

### Characterizing the F1 Population of Norton and Cabernet Sauvignon With the Identified Compounds

**FIGURE 7:**
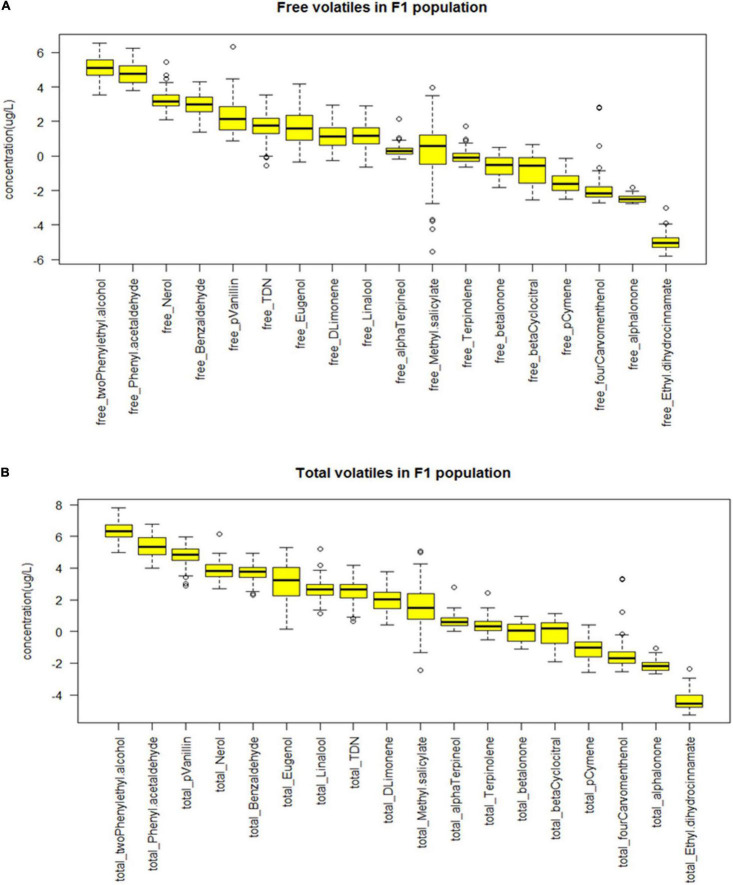
Boxplots demonstrating the variability of the **(A)** free and **(B)** total volatiles in F1 population cross from Norton and Cabernet Sauvignon (Cab). The concentrations of the volatiles in μg/L were log-transformed.

Using the identified compounds of interest from above, the F1 mapping population developed from the cross of Norton and Cabernet Sauvignon was screened for free and bound volatiles (Table 2). Fruits from 90 genotypes were quantified for 14 volatile compounds in the F1 population that exhibited clear differences in the Norton and Cabernet Sauvignon cultivars (Tables 1, 2). To identify useful potential phenotypes, we first identified the compounds that were different in the parents and the phenotype of those compounds in the progeny, i.e., F1 population. The boxplot showing the distribution of free and total volatiles in the F1 population is shown in Figures 7A,B. All the compounds exhibited a continuous variation in the progeny, i.e., the F1 population, which is typical for a polygenic inheritance as aroma compounds are complex and known to be governed by polygenes with small effects. We found many F1 genotypes that had a higher concentration of volatile compounds than the parents as well as many genotypes that had a lower concentration of compounds than the parents. In other words, transgressive segregation occurred in both directions. Aroma compounds exhibited different frequency distributions in the F1 population indicating different modes of inheritance (Supplementary Figure 2).

**TABLE 2:** Minimum, maximum, and mean of concentration of free and bound volatiles in μg/L in F1 population of Norton and Cabernet Sauvignon.

Volatile compounds	F1 population					
	Free volatiles			Total volatiles		
	Mean	Min	Max	Mean	Min	Max
2-Phenylethyl alcohol	206.47	33.84	693.03	673.43	149.02	2453.36
β-Phenyl acetaldehyde	142.33	45.28	517.16	265.01	53.54	858.97
Nerol	30.8	8.28	227.39	56.93	14.89	461.06
Benzaldehyde	23.64	4	73.3	46.42	10.13	138.42
Eugenol	9.65	0.72	65.59	42.1	1.17	200.85
TDN	6.92	0.57	34.42	15.85	1.92	64.19
D-Limonene	4.29	0.78	19.17	9.09	1.52	43.82
β-Linalool	4	0.53	18.45	18.23	3.17	179.07
Methyl salicylate	3.69	0	52.5	11.37	0.09	157.25
α-Terpineol	1.47	0.84	8.7	2.17	1.02	16.36
Terpinolene	1.06	0.52	5.51	1.73	0.6	11.29
β-Ionone	0.65	0.16	1.66	1.13	0.33	2.56
β-Cyclocitral	0.61	0.08	1.93	1.21	0.15	3.1
p-Cymene	0.27	0.08	0.87	0.41	0.07	1.51

## Discussion

A critical step in generating markers is identifying novel phenotypes (Schauer et al., 2006). As a step toward better markers for quality, it is critical to first understand the difference between the parents (Dunemann et al., 2009; Hernandez-Jimenez et al., 2013). Phenotypes can involve well understood and relatively easily measurable traits like vine vigor, yield, or cold hardiness (Reisch et al., 2012) as well as complex traits like flavor or aroma compounds. Flavor chemistry due to the thousands of analytes at play, with sometimes poorly understood perception is very complex (Ebeler and Thorngate, 2009). With wine, the fermentation adds further to the complexity of identifying chemicals of interest as the yeast convert precursors into new odor active compounds, sometimes with varying outcomes between industrial yeast strains or fermentation conditions (Callejon et al., 2012). In this study, we were able to streamline phenotype identification between the two cultivars by embracing commercially produced wines from around the world, rather than focusing on a limited region or breeding plot. We also chose to sample berries from different locations and varied the level of ripeness to identify the stable aroma phenotypes in the parents. This diverse sampling was added to system variability to identify stable aroma phenotypes regionally and annually. This is very significant as various viticultural conditions such as leaf shading, row orientation, and sun exposure can significantly impact the level of these compounds in berries (Kwasniewski et al., 2010; Gonzalez-Barreiro et al., 2015; Hernandez-Orte et al., 2015).

An untargeted metabolomics-based workflow followed by quantitation was used to study the understudied cultivar Norton. To the best of our knowledge, this is the first study that used an untargeted approach to profile the volatiles important in Norton wines and berries. This untargeted approach provided the ability to identify as many metabolites of potential interest as possible or without the requirement of pre-information about the compounds due to the comprehensive and unbiased nature of untargeted metabolomics (Awale et al., 2021). While metabolites provide phenotypic information about cells in response to different environmental and genetic changes, the genetics of the cultivar is the most significant factor impacting the volatile composition of grapes and wine (Ghaste et al., 2015). Our results strongly support that Norton and Cabernet Sauvignon wines are distinct from each other in their volatile composition. Norton wines had significantly higher concentrations of methyl salicylate, TDN, and *p*-cymene. Even with commercial wines from different wineries in the United States and Australian grape, genetics was the driving factor in aroma difference (Supplementary Table 6). This was in spite of us intentionally not controlling for location, yeast strain used, the winemaking process, or the use of different types of oak to maximize the diversity of wines to find only the most stable genotype-induced traits. The PCA scores plot of wine volatiles showed some overlap yet two clear groups between Norton and Cabernet Sauvignon wines (Supplementary Figure 1). The compounds that are found to overlap with wines from both cultivars are mostly esters such as methyl octanoate, ethyl nonanoate, and isoamyl hexanoate. These compounds are formed during the process of fermentation which would be expected to not have a strong cultivar influence (Figure 4C). Many other compounds also existed in wine from both cultivars, but at varied concentrations, supporting the hypothesis that the driving difference in aroma of some hybrid between *V. vinifera* counterparts is concentration-based versus unwanted impact odorants. Similarly, the berries used for free and bound volatiles analysis were sourced from different locations within Missouri and Virginia and harvested at different stages of ripening, and despite this intentional lack of controlling for field influence on quality clear and consistent cultivar differences were observed (Supplementary Table 4). The intent of using diverse samples from different years and regions was to identify those compounds that are the largest, most consistent drivers of difference between Norton and Cabernet Sauvignon and therefore are likely to be observed in fruit from their progeny regardless of the location or year. A controlled study between Norton and Cabernet Sauvignon may allow for the identification of additional traits and minimize the environmental variations such as soil, rainfall, water availability, sunshine, as well as variation in viticultural operations and winemaking practices. All these environmental, viticultural, and winemaking practices have been found to have a subtle but significant impact on the berry and wine volatile composition (Main and Morris, 2008; Hernandez-Orte et al., 2015; Alessandrini et al., 2017; Awale et al., 2021). Our results thus support that genetic differences were the most significant driver of aroma differences in the two cultivars (Ghaste et al., 2015), but also that the compounds driving these differences can be identified in commercial samples helping quickly guide further investigation.

A critical aspect of any untargeted study is to understand and adjust for run-to-run variability in the data. This may be caused by variation elements such as instrument sensitivity, sample preparation, or sample degradation while waiting for analysis. One common way to do this is through the use of quality control samples, which are often composite samples created from an equal volume of all analysis samples to be run. In running these periodically, it is then possible to measure the coefficient of variance among these samples, either re-running sample blocks that fall outside of a given tolerance or using this variability to normalize the data of experimental samples (Mizuno et al., 2017). Samples can also be randomized to ensure any signal changes due to temporal versus true sample differences are not inadvertently picked up as a treatment difference, thus protecting from type one error. With or without sample randomization, it is also possible to control for variance to some degree by normalizing the data by the sum of the areas or by use of endogenous “house-keeping” compounds or metabolic markers that stay at a fairly constant concentration (Wu and Li, 2016). While these methods of normalization have their uses, they do also add a degree of uncertainty to the analysis as you are in fact normalizing for aspects that you do not know to be the same. In this work, we opted for another option, whereby an internal standard is added, even during untargeted analysis. This is standard practice with targeted work, as it adds the benefit of a known point of normalization, which can be utilized even in the feature characterization phase (Mizuno et al., 2017). Additionally, variance from the mean can be monitored in every sample, accounting for possible run problems with a sample, whereas QC samples will not differentiate between block or sample variance problems. In addition, all samples were randomized and compared only within a given run block to avoid any influence of instrument drift not accounted for by the internal standard. Further findings were compared across different sample sets and types (wine, berry, and total berry), often finding similar features of interest. With key findings taken through to semi-quantification with authentic standards, making type I error was very unlikely.

Most untargeted metabolomics studies end with compound identification (Schrimpe-Rutledge et al., 2016). Our study goes a step ahead with the quantification of the identified compounds as for breeding and genetic analysis, and the presence or absence of compounds in the parents does not have a greater impact unless they have concentration differences in the parents. Compounds that are present in higher concentrations in one cultivar and lower in the other can be used as a phenotype and maker of the cultivar. Being a hybrid grape cultivar, very limited studies have looked at the quantitative measure of volatile compounds in Norton. We did not find any compound that was present in one cultivar and absent in the other, but there was a huge variation in the concentration of some compounds (Table 1). The presence of some compounds above the odor threshold in Norton such as eugenol (55.8 μg/L), and the presence well below the odor threshold (3 μg/L) in Cabernet Sauvignon (0.425 μg/L) might be responsible for the distinct aroma in the two cultivars. Eugenol is a phenylpropanoid derived volatile compound which is described as having a pungent, minty, and clove oil aroma widespread in many plant species. Eugenol is more abundant in non-vinifera species, mostly in its bound form (Sun et al., 2011; Ghaste et al., 2015). However, our results indicated that Norton had higher free eugenol than its bound fraction, which might be due to multiple enzyme activities (esterase, oxidase in addition to glucosidase) of the Rapidase enzyme (Hampel et al., 2014). The presence of eugenol at such higher concentrations in Norton might explain the minty aroma in some Norton wines.

Of note was that methyl salicylate was found to be higher in Norton in free, total, and wine volatiles (Figure 5). Although not present above the odor threshold in Norton, some of the F1 progenies had higher concentrations than the odor threshold. Methyl salicylate is a methylated form of stress hormone salicylic acid (SA) associated with biotic stress caused by obligate pathogens. SA is a benzoic acid derivative, generally involved in the activation of defense responses against biotrophic (which keeps the cell alive) and hemi-biotrophic (which initially keep the cell alive but kill them at later stages) pathogens. Methyl ester of SA, methyl salicylate, can act as volatile systemic acquired resistance (SAR) inducing signals transmitted to distant plant parts or even the surrounding plant parts. SA being immobile, methyl salicylate has been known as one of the mobile signaling molecules. Methylation inactivates SA while increasing its membrane permeability and volatility, allowing more effective long-distance transport of the defense signal (Dempsey et al., 2011). Earlier studies have found that Norton leaves accumulate high levels of SA and SA-related defense genes in comparison to Cabernet Sauvignon, which may have contributed to a robust innate defense system against pathogens in Norton grape (Fung et al., 2008). The higher proportion of methyl salicylate in grapes and wines of Norton shows the presence of a defense mechanism and SAR, which makes these berries resistant to many grape pathogens. This may be the reason why Norton grapes are resistant to many disease pathogens, in comparison to Cabernet Sauvignon, which had lower levels of methyl salicylate. While glycosylated methyl salicylate had been found in some *vinifera* berries (Carlin et al., 2019), our results indicated that most of the methyl salicylate was present in free form. The reason for this might be the prevalence of higher free form in Norton grapes, which need to be investigated further, or might be due to esterase side activity of the Rapidase enzyme in addition to glycosidase activity (Hampel et al., 2014). The frequency distribution of methyl salicylate in the F1 population was continuous, suggesting the regulation of multiple genes, ranging up to 52.50 μg/L of free volatiles and 157.25 μg/L of total volatiles.

The aroma compound β-damascenone was found to be two times more abundant in Norton than in Cabernet Sauvignon for all free, total, and wine volatiles, while present above the odor threshold in both cultivars (Figure 6 and Table 1). In the F1 population, free β-damascenone demonstrated continuous distribution, with some F1’s having concentrations up to 42 μg/L. β-Damascenone is a very important carotenoid-derived C_13_-norisoprenoid that is noted for its baked apple, honey, and fruity flavor. It is also a flavor enhancer, impacting the perception and odor threshold of other compounds (Pineau et al., 2007). Typically, in *vinifera* red wines, β-damascenone is present at or below the odor threshold (1–2 μg/L) and was found to have an indirect impact on red wine aroma by enhancing the threshold of fruity esters (ethyl cinnamate and ethyl caproate) and decreasing the green bell pepper aroma (Pineau et al., 2007; Sefton et al., 2011). Earlier studies have reported a higher amount of β-damascenone in non-vinifera cultivars, up to 30 μg/L in St. Croix (Slegers et al., 2015, 2017). The higher amount of this compound in grapes and wines is often correlated with more fruity flavors (Sefton et al., 2011). This suggests that an elevated amount of this compound might be responsible for the overwhelming fruity aroma of Norton grapes and wines.

Free TDN was found to be higher in Norton than in Cabernet Sauvignon with mean levels of 7.35 and 2.66 μg/L, respectively (Table 1). Also, a C_13_ norisoprenoid derived from carotenoid degradation, TDN, has a very low sensory threshold (2 μg/L) and thus can impart negative attributes to wine at higher concentrations (Sacks et al., 2012). It is a characteristic varietal character of aged Riesling wine (concentration up to 50 μg/L); however it is generally found lower than its threshold in other vinifera cultivars, with exception of being found at 6.4 μg/L in Cabernet Franc (Kwasniewski et al., 2010; Sacks et al., 2012). This compound has not been reported in interspecific hybrids in previous studies; however due to the lack of commercial standard availability, investigations of this compound are limited as compared to other wine aromas. In the F1 population of Norton by Cabernet Sauvignon cross, the concentration of TDN was found to be higher than that of any parents, averaging 6.89 μg/L for free and 15.70 μg/L for total volatiles. We observed some F1 genotypes with much higher concentrations (up to 64 μg/L total TDN), showing transgressive segregation. It should be noted that while Cabernet Sauvignon had TDN concentrations lower than the odor threshold, Cabernet Franc, a known TDN produce, is one of Cabernet Sauvignon’s parents.

Monoterpenes such as β-caryophyllene, β-linalool, *p*-cymene, and nerol were found to be more abundant in Norton than Cabernet Sauvignon. Terpenes, also known as isoprenoids are one of the important secondary metabolites in plants important for the plant’s resistance to diseases and pests. They are an important volatile constituent in grape berries present in free and bound form and biosynthesized from the primary metabolites through the mevalonic pathway and methylerythritol pathway in cytosol and chloroplast respectively. It has been found that the *de novo* synthesis of monoterpenes and sesquiterpenes occurs *via* the octadecanoid signaling cascade using methyl jasmonate which is involved in plant defense (Schwab and Wüst, 2015). The abundance of monoterpenes and sesquiterpenes in Norton was higher than Cabernet Sauvignon grapes and wines, which indicated the probable role of regulation of stress hormone, jasmonic acid, and disease resistance nature of Norton.

While out of the scope of this study, it has been demonstrated by others that precursors in the fruit beyond glycosides have important impacts on wine volatiles (Ebeler and Thorngate, 2009). These includes esters, alcohols, phenols, and aliphatic acids (Liu et al., 2017). As these compounds are of interest, we included them in the commercial wine analysis finding several compounds that differ by cultivar. This has also been found by others that have done varietals difference in wine aroma and points to the additional wealth of aroma that may be taken forward through primary and/or malolactic fermentation (Yang et al., 2009; Liu et al., 2017).

The F1 population between Norton and Cabernet Sauvignon has been used successfully to find quantitative trait loci (QTLs) related to various disease resistance (Adhikari et al., 2014; Sapkota et al., 2015, 2019). Recently, the same population used in our work was studied to uncover the genetic basis of berry organic acids, a complex trait, led to the identification of multiple QTLS (Negus et al., 2021). Similarly, our study in the same F1 population identified the marker aroma compounds such as methyl salicylate, TDN, and β-damascenone as complex quantitative traits, indicating polygenic inheritance (Figure 7 and Supplementary Figure 2). Although aroma in grapes and wine is a highly appreciated organoleptic attribute, little is known about the inheritance patterns of the aroma traits and the main compounds associated with it. Few genetic studies have been done to uncover the genetic regions underlying the development and formation of some aroma compounds in apples (Dunemann et al., 2009; Vogt et al., 2013) and grapes (Doligez et al., 2006; Battilana et al., 2009; Guillaumie et al., 2013) and have demonstrated variability and heritability of individual aroma compounds. Few genetic studies in grapes have focused on using QTL analysis that identified QTLs for muscat flavor in the three linkage groups (Doligez et al., 2006) and IBMP in the linkage groups 2 and 3 (Guillaumie et al., 2013) and found to be governed by multiple complementary genes and modifier genes. While mapping aroma traits are known to be complicated, this is in part because the phenotyping of aroma compounds in any breeding population is difficult as aromas are impacted by environmental factors such as climate, season, vintage, and location. Despite the innate complexity, through the use of robust traits, we found that most of the free and total aroma compounds exhibited a distribution in the F1 population which indicated that they are under genetic control. The levels of compounds in the F1 population generally located within and outside the range given by the two parental cultivars show transgressive segregation (Figure 6). The frequency distributions in the F1 population for individual aroma compound is different indicating different modes of inheritance. This might be due to the complex biochemical pathways from which volatiles are derived and varied ripening rates of the progeny. As grapes are heterogenous, the F1 genotypes have a good probability of showing transgressive segregation which were not seen in their parents but seen in the grandparents. For example, Cabernet Franc as one of the parents of Cabernet Sauvignon, possess some compounds which were not present or present below odor threshold in Cabernet Sauvignon but can now appear in its grandchildren, an example being TDN. Thus, aroma compounds can be used as stable phenotypes and further could be exploited to uncover its genetic architecture using approaches like QTL mapping. Identifying QTLs will help to cumulate favorable alleles at several loci to reach stable aroma phenotype that can be used in breeding new grape cultivars that are both disease resistant and desired aroma character.

Grapes, being a perennial crop, have a long juvenile period. It is both expensive and time-consuming to maintain and phenotype a breeding population as it requires a lot of labor and resources (Reisch et al., 2012). Therefore, it is even more essential to consider the parental volatile profile before making a cross so that the breeding population can be phenotyped using the aroma markers. Our study strongly demonstrated the value by using the aroma markers in the F1 population. This might improve the consumer acceptance of new environmentally sustainable hybrid cultivars of grapevines by providing the ability to select for the desired aroma phenotypes by breeders. The development of molecular markers linked to genes for key aroma compounds will be an important step toward a future marker-assisted breeding (Tikunov et al., 2005).

## Conclusion

This work demonstrates that an untargeted metabolomics-based workflow paired with samples acquired from a supermarket can allow the identification of stable phenotypes of interest. Our study has demonstrated that Norton and Cabernet Sauvignon wines had significant differences in their aroma profile that could be leveraged for future genetic study. We used an integrated metabolomics workflow and narrowed down thousands of features to identify 33 important aroma compounds in Norton and Cabernet Sauvignon. Our results uncovered around half of the identified compounds used as aroma phenotype traits in the F1 breeding population and demonstrated extensive variability and genetic segregation. Future investigation should focus on dissecting the genetic architecture of these aroma compounds to enable marker-assisted selection and breeding grapevines for biotic and abiotic stress resistance with desired aroma profile.

## Data Availability Statement

The original contributions presented in this study are included in the article/Supplementary Material, further inquiries can be directed to the corresponding author.

## Author Contributions

MA and CL: data collection and instrumental analysis. MA: data curation analysis and writing—original draft preparation. MK: conceptualization and funding acquisition. MA and MK: writing—review and editing. MK: supervision. All authors have read and agreed to the published version of the manuscript.

## Conflict of Interest

The authors declare that the research was conducted in the absence of any commercial or financial relationships that could be construed as a potential conflict of interest.

## Publisher’s Note

All claims expressed in this article are solely those of the authors and do not necessarily represent those of their affiliated organizations, or those of the publisher, the editors and the reviewers. Any product that may be evaluated in this article, or claim that may be made by its manufacturer, is not guaranteed or endorsed by the publisher.

## Footnotes

1
https://xcmsonline.scripps.edu/landing_page.php?pgcontent=mainPage

## Supplementary Material

Supplementary Figure 1 Principal components analysis scores map of the model obtained for **(A,B)** free, **(C,D)** total, and **(E,F)** wine volatiles indicating the distinct separation of Norton and Cabernet Sauvignon for wine volatiles using quantitative data and the biplot showing volatiles contributing to the differences in Norton and Cab wine volatiles.

Supplementary Figure 2 Frequency distribution of **(A)** free eugenol, **(B)** total eugenol, **(C)** free methyl Salicylate, and **(D)** total methyl salicylate in F1 population of Norton and Cabernet Sauvignon. The concentration values in mg/L were log-transformed.
