# Supplementary figures and images for: Generating Novel Aroma Phenotypes Using Commercial Wine Samples to Characterize an F1 Population
Source: Front Plant Sci. 2022 Jun 21;13:894492. doi: 10.3389/fpls.2022.894492 (PMC9253817; doi:10.3389/fpls.2022.894492)

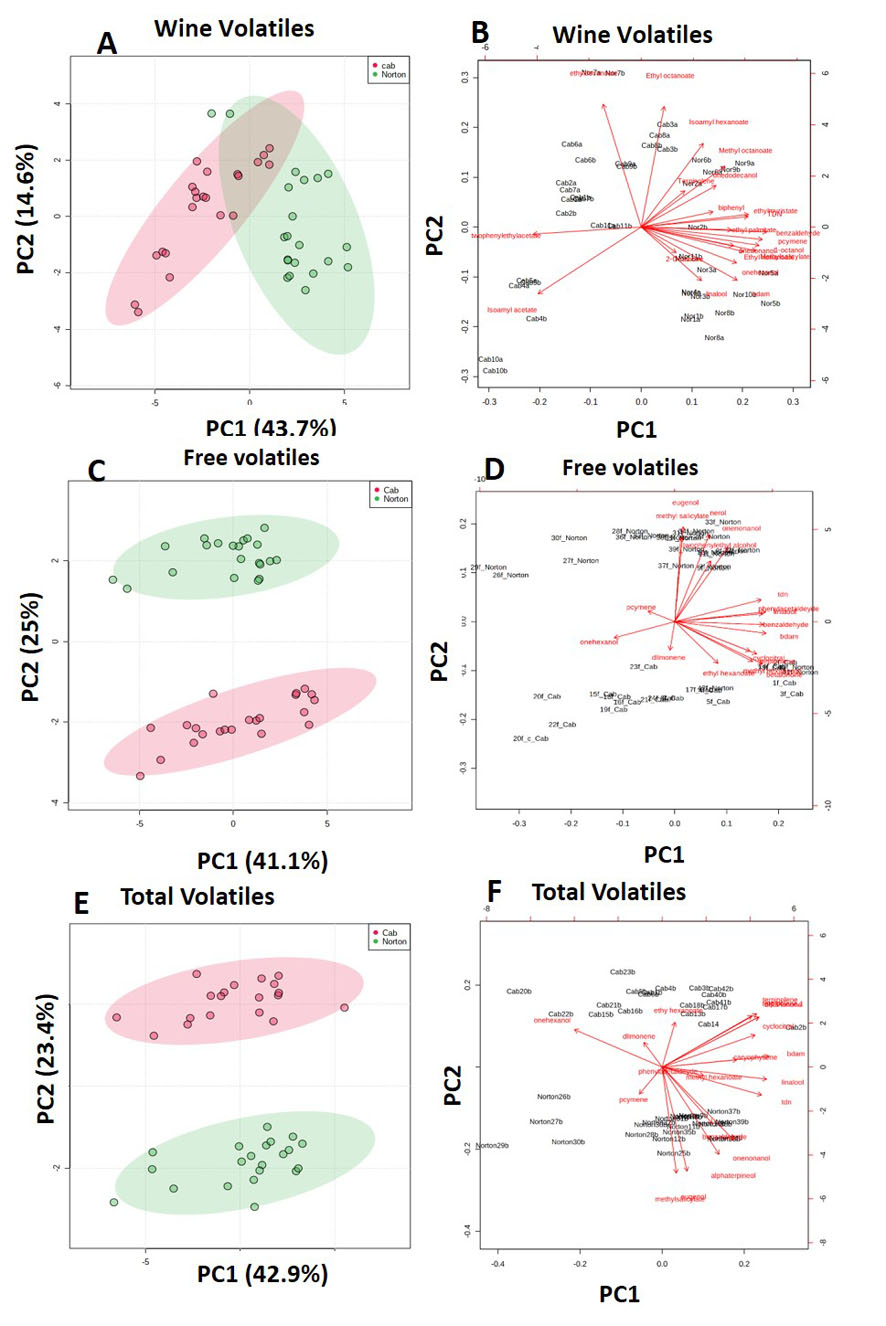

Supplement: Supplementary Figure 1 — Principal components analysis scores map of the model obtained for (A,B) free, (C,D) total, and (E,F) wine volatiles indicating the distinct separation of Norton and Cabernet Sauvignon for wine volatiles using quantitative data and the biplot showing volatiles contributing to the differences in Norton and Cab wine volatiles. [file Image_1.JPEG]

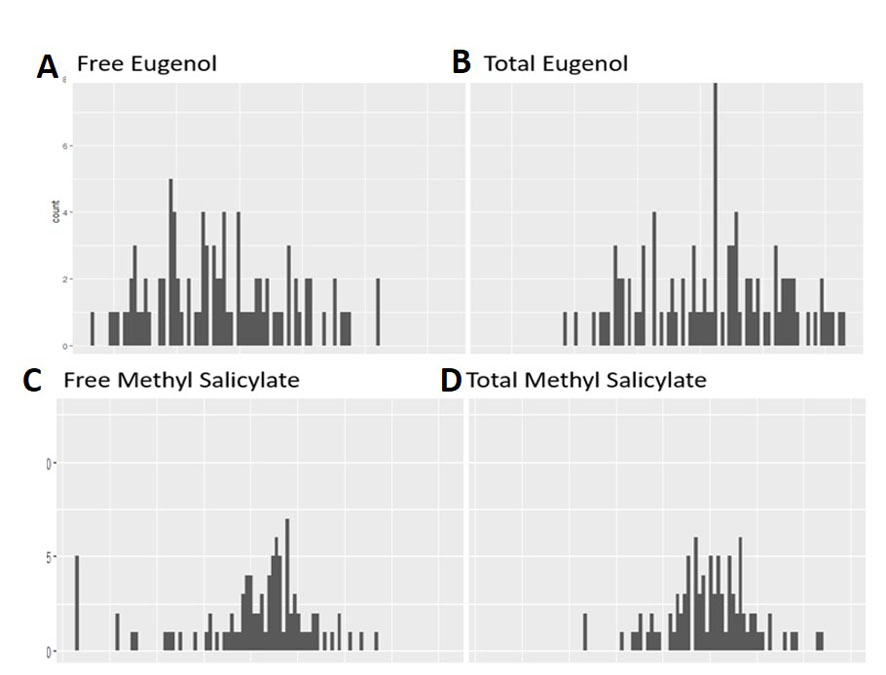

Supplement: Supplementary Figure 2 — Frequency distribution of (A) free eugenol, (B) total eugenol, (C) free methyl Salicylate, and (D) total methyl salicylate in F1 population of Norton and Cabernet Sauvignon. The concentration values in mg/L were log-transformed. [file Image_2.JPEG]
